# Efficacy and safety of acupuncture in the treatment of Meniere’s disease: a systematic review and meta-analysis

**DOI:** 10.3389/fmed.2024.1463821

**Published:** 2024-12-11

**Authors:** Mingjie Tang, Yinghong Li, Man Lu, Tianchen Zhang, Yanhao Ge, Jie Han, Jiqin Tang, Zhaoming Chen

**Affiliations:** ^1^Nanjing University of Chinese Medicine, Nanjing, Jiangsu, China; ^2^Nanjing Hospital of Chinese Medicine Affiliated to Nanjing University of Chinese Medicine, Nanjing University of Chinese Medicine, Nanjing, Jiangsu, China; ^3^College of Rehabilitation Medicine, Shandong University of Traditional Chinese Medicine, Jinan, Shandong, China

**Keywords:** acupuncture, systematic review, meta-analysis, Meniere’s disease, vertigo

## Abstract

**Background:**

Meniere’s disease (MD) is an idiopathic chronic inner ear disease that seriously impacts patients’ physical and mental health. Medications may be effective for a proportion of patients, and additional effective treatments are still needed. This review aimed to evaluate the efficacy of acupuncture treatment for MD.

**Methods:**

Eight databases were systematically searched from their inception to June 1, 2024, to identify randomized clinical trials on acupuncture treatment for MD. The Cochrane Risk of Bias 2.0 tool was used to assess the risk of bias in included studies, and meta-analysis was conducted by RevMan 5.4 and Stata 16.0 software.

**Results:**

Six studies were included in this review. The treatment group received acupuncture or acupuncture combined with Western medicine, while the control group was treated with Western medicine. The treatment group was superior to the control group in terms of efficacy rate (RR: 1.20; 95% CI: 1.11–1.29; *p* < 0.0001). The treatment group reduced dizziness handicap inventory (DHI) (MD: 6.94; 95% CI: 1.58–12.30; *p* = 0.01), tinnitus handicap inventory (THI) (MD: 6.52; 95% CI: 0.77–12.27; *p* = 0.03), stuffy ear visual analog scale (VAS) (MD: 0.87; 95% CI: 0.54–1.20; *p* < 0.0001) and pure tone audiometry score (MD: 6.57; 95% CI: 5.62–7.51; *p* < 0.0001) to a greater degree than those of the control group. There were some methodological shortcomings in the included studies, including failure to implement blinding, inappropriate outcome measures, and heterogeneity of clinical interventions, such as selected acupoints, acupuncture sessions, and therapist techniques.

**Conclusion:**

Acupuncture may improve the symptoms of vertigo, tinnitus, ear fullness and hearing loss in patients with MD. However, due to the lack of literature included in this study and methodological weaknesses like randomization, blinding, and clinical heterogeneity, more well-designed long-term follow-up RCTs are needed to evaluate the efficacy and safety of acupuncture.

**Systematic Review Registration:**

https://www.crd.york.ac.uk/PROSPERO/(CRD42024549261).

## 1 Introduction

Meniere’s disease (MD) is a chronic inner ear disorder characterized by the abnormal accumulation of endolymph within the labyrinth. Clinically, MD manifests as recurrent episodes of vertigo, fluctuating hearing loss, tinnitus, and a sensation of fullness in the affected ear (1). Literature reports show that the prevalence of MD varies widely, ranging from 17 to 513 per 100,000 population, with an increasing trend in Meniere’s disease incidence in the last 10 years (2). It is estimated that approximately 50% of patients diagnosed with MD also experience comorbid depression (3). Additionally, a study found that between 78 and 89% of people with MD experience constraints in their everyday activities, employment, and travel, which significantly affects their general quality of life (4).

The exact cause of MD is still unknown. Unbalances in endolymphatic homeostasis may arise from the dysregulation of hormone secretion, including that of aquaporins, ion channels, adrenaline, and anti-diuretic hormone, according to recent study on etiological causes and pathological processes (5). To date, there is still no gold standard for treatment guidelines for MD. Currently, the main treatments for MD include dietary therapy, betahistine, diuretics, transtympanic corticosteroid injection, transtympanic gentamicin, tympanic low-pressure pulsation therapy, surgery, and vestibular rehabilitation (6). It should be noted that not all patients respond positively to the drugs. Despite taking these drugs, some people may not acquire adequate control, and other medications, like betahistine, can have negative side effects, such as gastrointestinal responses and abnormalities of the neurological system (mostly headaches) (7, 8). Endolymphatic sac decompression is one of the most frequently utilized surgical techniques for the management of vertigo. However, due to the inherent limitations of this approach and the findings of the Cochrane Systematic Review, which suggested that endolymphatic sac surgery may not be a highly efficacious intervention for MD, there is a necessity to reassess its role in clinical practice (9). There is a necessity for the development of more effective and safer complementary and alternative therapies for the treatment of MD. Complementary and alternative therapies refer to a wide range of non-conventional medical practices used in conjunction with, or instead of, conventional medicine, such as western medicine and surgery (10, 11). These practices may include acupuncture, herbal medicine, dietary supplements, and other non-traditional methods.

Acupuncture, as an important component of complementary and alternative therapies, has been widely used in the clinical treatment of MD (12, 13). A combination of scalp clustering acupuncture and ear acupressure has been demonstrated to alleviate the most significant symptoms associated with MD to a certain extent, including vertigo and tinnitus (14). Acupuncture of local points may provide relief of vertigo symptoms by increasing the blood supply to the vertebrobasilar system and promoting blood circulation to the brain, or by improving vestibular function and promoting the recovery of inner ear function (15). Therefore, we conducted this systematic review of randomized controlled trials (RCTs) to further assess the effect of acupuncture for MD.

## 2 Materials and methods

### 2.1 Registration

The protocol for this systematic review and meta-analysis was registered in PROSPERO (CRD42024549261) and was in accordance with the Preferred Reporting Items for Systematic Reviews and Meta-Analyses (PRISMA) guidelines (16).

### 2.2 Study design

We only included randomized controlled trials (RCTs). We did not place any restrictions on language.

### 2.3 Research subjects

The patients met the diagnostic criteria for Meniere’s disease in accordance with the European Academy of Otology and Neurotology, and American Academy of Otolaryngology-head and neck surgery guidelines (17). We do not have any restrictions on age or gender.

### 2.4 Interventions

The treatment group was treated with acupuncture-related therapy including manual acupuncture, electroacupuncture, auricular point sticking, warm needling, auricular acupuncture, plum blossom needle, bloodletting therapy, or acupuncture combined with Western medicine. The control group received Western medicine such as betahistine and mecobalamin.

### 2.5 Outcome indicators

The primary outcome indicator was the efficacy rate. The secondary outcome indicators were the dizziness handicap inventory (DHI), tinnitus handicap inventory (THI), stuffy ear visual analog scale (VAS), and pure tone audiometry score.

### 2.6 Exclusion criteria

We excluded studies based on the following characteristics: (1) studies with other Chinese medicine treatments in any group, such as Chinese herbal medicine, Chinese Patent Medicine, and massage; (2) studies with obvious errors in the literature data, research methodology, design methodology, statistical methodology or the data records are not well documented; (3) lack of outcome indicators; and (4) duplicate studies.

### 2.7 Literature search strategy

We searched eight databases from their inception to June 1, 2024. Two researchers (TMJ and LYH) independently searched PubMed, EMBASE, Web of Science, Cochrane Library, Chinese National Knowledge Infrastructure (CNKI), Wanfang Database, Chinese Science and Technique Journals Database (VIP) and SinoMed. The search terms were “Meniere’s disease” “Meniere’s Syndrome” “Meniere disease” “acupuncture therapy” “acupuncture” “acupoint” “electroacupuncture” “randomized controlled trial” and related terms. Detailed search strategies are shown in Supplementary material 1.

### 2.8 Literature screening and data extraction

The literature was imported into Endnote X9 software from various databases. After automatic weight removal, two researchers (TMJ and LYH) conducted preliminary screening by reading all the titles and abstracts. Subsequently, the researchers carefully read the full text of the screened literature and selected the qualified literature based on the inclusion criteria. During the full-text screening process, relevant information will be extracted, the researchers extracted information and entered it into the “information extraction form.” This form included authors, year of publication, sample size, demographic baseline, intervention, duration of treatment, outcomes, and adverse events. Any uncertainties or disputes were resolved through discussion or consultation with a third researcher (LM).

We extracted the treatment details given the Revised Standards for Reporting Interventions in Clinical Trials of Acupuncture (STRICTA) (18). The STRICTA checklist contains six main items: (1) Acupuncture rationale, (2) Details of acupuncture, (3) Treatment plan, (4) Other interventions, (5) Therapist’s background, and (6) Control or comparator intervention.

### 2.9 Risk of bias assessment

The methodological quality of the included studies was independently assessed by two researchers (GYH and ZTC) using the Cochrane Risk of Bias Tool 2.0 (RoB 2.0) (19). It contains six aspects: generation of randomization sequences, deviations from the intended interventions, missing outcome data, measurement of the outcome, selective reporting of outcomes, and overall bias. The assessment was classified into three groups: “low,” “high,” and “some concerns.” Any disagreements or disputes were resolved through discussion with a third researcher (LM).

### 2.10 Statistical analysis

We used Review Manager 5.4 and Stata 16.0 software for statistical analysis. If *I*^2^ ≤ 50%, and *p* ≥ 0.1, it indicates low heterogeneity between studies. If *I*^2^>50%, and *p* < 0.1, it indicates substantial heterogeneity between studies, which may be due to clinical heterogeneity or subgroup analysis. Otherwise, the source of heterogeneity was further analyzed. For continuous variables, the mean difference (MD) was employed as the effect indicator when described by the same scale, whereas the standardized mean difference (SMD) was utilized as the effect indicator when described by different scales. For dichotomous variables, we used the risk ratio (RR). Efficacy rate was calculated as RR, which measures the treatment effect by calculating the ratio of event rates between the treatment group and the control group. We calculated the confidence intervals (CI) at 95%. Funnel plots and Egger’s test were employed to assess the potential for publication bias. The asymmetric funnel plot or a probability value (*p* < 0.05) of Egger’s test indicated that there was significant publication bias.

### 2.11 Quality of evidence

The quality of evidence was evaluated using the Grading of Recommendations, Assessment, Development, and Evaluations (GRADE) system (20) for each outcome. It contains five aspects: risk of bias, imprecision, indirectness, inconsistency, and publication bias.

## 3 Results

### 3.1 Literature retrieval results

**Figure 1:**
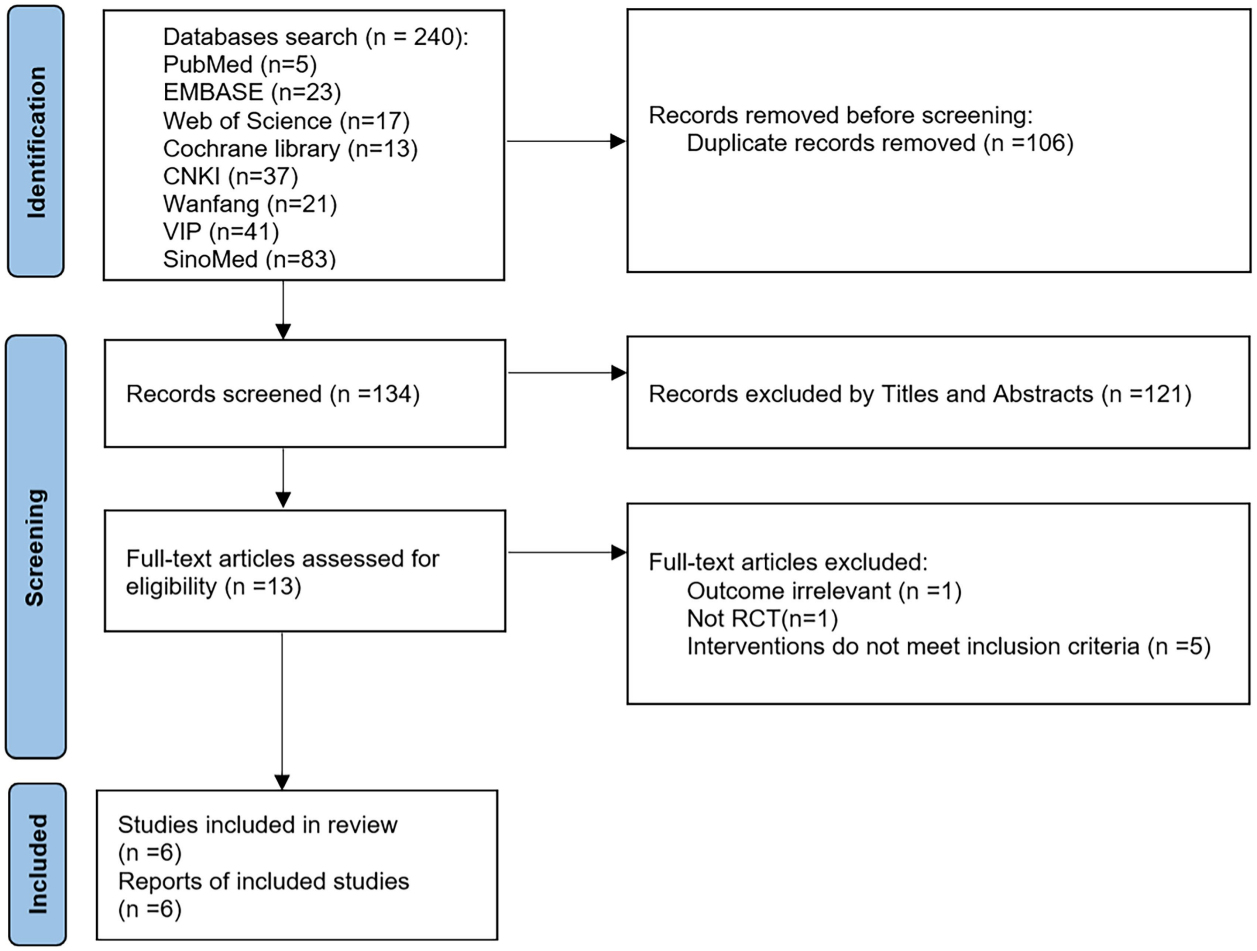
PRISMA flow diagram.

A total of 240 articles were retrieved using the search strategy and then 106 duplicates were excluded. Then 121 articles were excluded by reading the title/abstract and 7 articles by reading the full text (1 with irrelevant outcomes, 1 non-RCT, and 5 with discrepancies in interventions). Finally, 6 studies (21–26) were included. The screening process is shown in Figure 1.

### 3.2 Characteristics of the literature

The total sample size of patients included in the 6 studies (21–26) was 494 cases, with 248 in the treatment group and 246 in the control group. All six studies (21–26) were conducted in China and published in Chinese. Six studies (21–26) reported the efficacy rate, and two (23, 26) of them compared and analyzed the efficacy of the two groups in terms of dizziness, hearing, and mobility, and we took the average of these three aspects to calculate the total efficacy rate. Three studies (22, 23, 26) reported DHI, THI, VAS, and pure tone audiometry score. A total of five RCTs (22–26) employed MA in conjunction with Western medicine, in comparison to Western medicine alone. Conversely, only one RCT (21) compared MA with Western medicine. The characteristics of each included study in Table 1. The completeness of reporting based on STRICTA guidelines and therapeutic acupoints for each study are shown in Supplementary materials 2, 3. Details of needling are presented in Supplementary material 4.

**Table 1:** Characteristics of RCTs of acupuncture for the treatment of Meniere’s disease.

Author, year	Sample size	Age (year)		Female (%)	Duration of disease (y)	Interventions		Course of treatment	Outcomes	Adverse events
		T	C			T	C			
Ai2013	54/49	36.00 ± 7.25	39.00 ± 6.75	62.1%	NR	MA	Betahistine, niacin, vitamin B_1_, B_6_, promethazine	10-30d	a	NR
Ding2021	58/58	69.48 ± 3.45	69.31 ± 3.39	69.8%	11.01 ± 2.35/11.13 ± 2.30	MA+ Betahistine, mecobalamin	Betahistine, mecobalamin	3 m	abcde	NR
Ha2023	30/30	45.22 ± 10.65	46.18 ± 11.89	55.0%	3.76 ± 1.98/3.88 ± 2.19	MA+ Betahistine	Betahistine	4w	abcde	0/0
Mao2014	30/30	35.00 ± 6.00	38.00 ± 5.75	41.7%	NR	MA+ Betahistine	Betahistine	7d	a	NR
Wang2011	40/40	NR	NR	52.5%	NR	MA + Betahistine, salvia miltiorrhiza injection, flunarizine, anisodamine, hydrochlorothiazide	Betahistine, salvia miltiorrhiza injection, flunarizine, anisodamine, hydrochlorothiazide	6w	a	NR
Wu2018	36/39	46.00 ± 9.00	44.00 ± 11.00	54.7%	6.9 ± 3.8/7.7 ± 4.6	MA+ Betahistine, mecobalamin	Betahistine, mecobalamin	12w	abcde	NR

C, control group; d, day; m, month; MA, manual acupuncture; NR, not reported; T, treatment group; w, week; a, efficacy rate; b, DHI; c, THI; d, VAS; e, pure tone audiometry score.

### 3.3 Risk of bias

**Figure 2:**
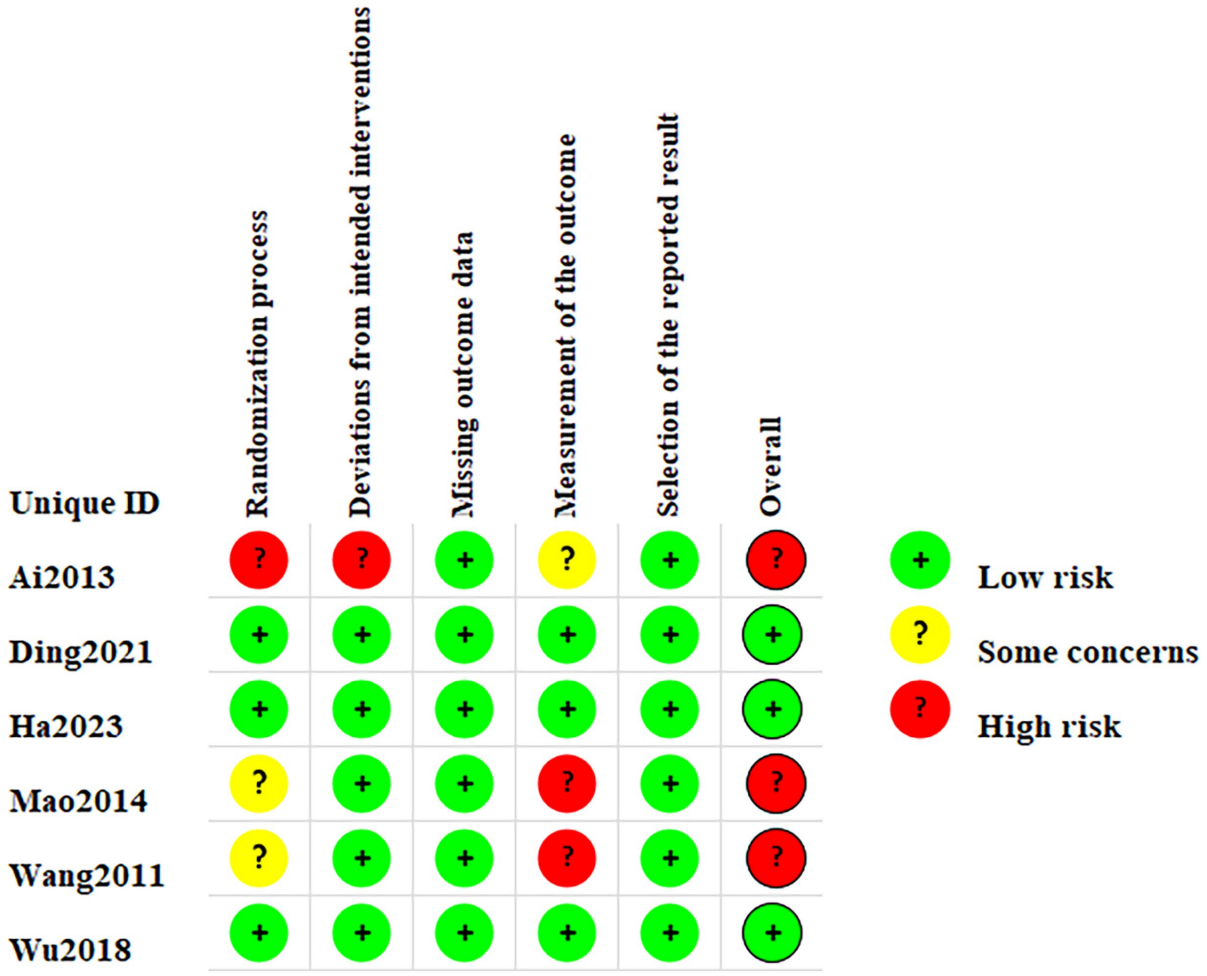
Risk of bias 2.0 ratings for included studies.

Two RCTs (22, 26) used the random number table method and one RCT (23) used the single-blind method thus leading to low risk during randomization. The remaining two RCTs (24, 25) did not provide any further information regarding the randomization methods employed leading to unclear risk of bias, and one RCT (21) without randomization grouping led to high risk of bias. One RCT (21) may have deviations from established intervention leading to high risk of bias. Two RCTs (24, 25) may have inappropriate outcome measures and were therefore classified as high risk of bias. Measurements from the RCT (21) may receive influences from the person who measured them and be classified as unclear risk of bias. Figure 2 summarizes the risk of bias in the 6 articles.

### 3.4 Certainty of evidence (GRADE)

Results of the GRADE evidence quality rating from very low to moderate are shown in Table 2.

**Table 2:** Summary of evidence quality for outcomes based on GRADE system.

Outcomes (No. of studies/patients)	Study design	Risk of bias	Inconsistency	Indirectness	Imprecision	Publication bias	Availability of upgrade conditions	Effect (95%CI); *I*^2^	Overall certainty of evidence
Efficacy rate (6/494)	RCT	serious^a^	not serious	not serious	serious^c^	serious^d^	None	RR:1.20(1.11,1.29); 0%	⊕○○○ Very low
DHI (3/251)	RCT	not serious	serious^b^	not serious	serious^c^	serious^d^	None	MD:6.94(1.58,12.30); 92%	⊕○○○ Very low
THI (3/251)	RCT	not serious	serious^b^	not serious	serious^c^	serious^d^	None	MD:6.52(0.77,12.27); 95%	⊕○○○ Very low
VAS (3/251)	RCT	not serious	not serious	not serious	serious^c^	serious^d^	None	MD:0.87(0.54, 1.20); 0%	⊕⊕○○ Low
Pure tone audiometry score (3/251)	RCT	not serious	not serious	not serious	not serious	serious^d^	None	MD:6.57(5.62,7.51); 0%	⊕⊕⊕○ Moderate

DHI, dizziness handicap inventory; THI, tinnitus handicap inventory; VAS, stuffy ear visual analog scale; RCT, randomized controlled trial; CI, confidence interval.

Explanations:

^a: downgrade one level: Missing blinding and randomization in some studies.^

^b^: downgrade one level: Heterogeneity between studies cannot be eliminated (*I*^2^ > 50%).

^c: downgrade one level: Confidence interval is too wide.^

^d: downgrade one level: Language limitations and small sample size may lead to publication bias.^

High certainty: We are very confident that the true effect is close to the estimated effect.

Moderate certainty: We are moderately confident in the effect estimate: the true value is probably close to the estimate, but there is still a possibility that they are different.

Low certainty: Limited confidence in effect estimation: true values are likely to be substantially different from estimated values.

Very low certainty: We have little confidence in the effect estimate: the true value is likely to be very different from the estimate.

### 3.5 Main findings

#### 3.5.1 Efficacy rate

**Figure 3:**
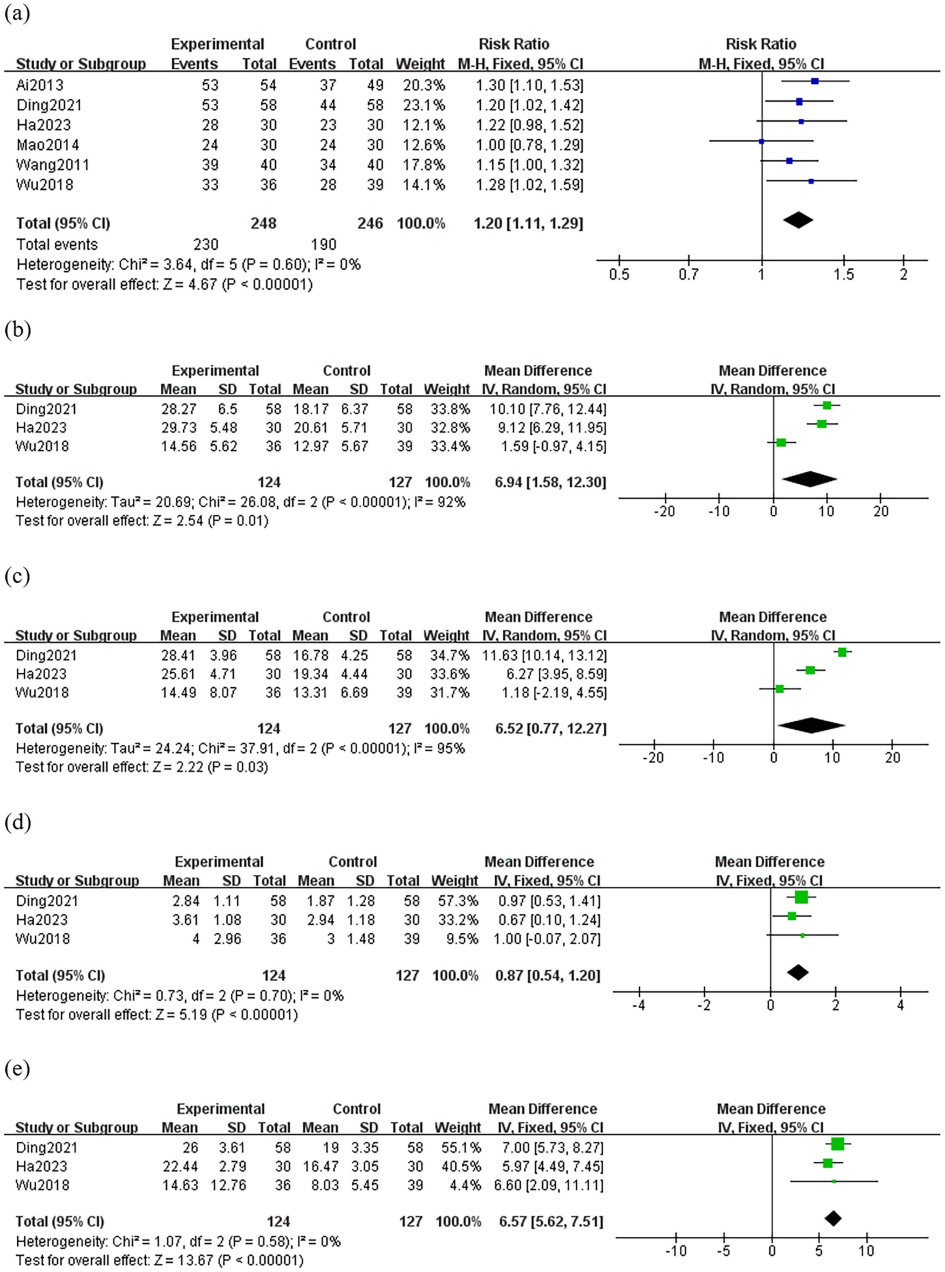
Forest plot comparing the treatment efficacy between the treatment group and the control group. Experimental group: treatment group; **(A)** efficacy rate **(B)** DHI **(C)** THI **(D)** VAS **(E)** pure tone audiometry score.

Six studies (21–26) compared the efficacy rate between groups. There was no significant heterogeneity among the studies in the efficacy rate (*p* = 0.60, *I*^2^ = 0%), so the fixed-effect model was used for the meta-analysis. The results showed that the treatment group was superior to the control group in terms of efficacy rate (RR: 1.20; 95% CI: 1.11–1.29; *p* < 0.0001) (Figure 3A).

#### 3.5.2 DHI

Three studies (22, 23, 26) compared the DHI between groups, and the random-effects model was used because of the high heterogeneity among the studies (*p* < 0.0001, *I*^2^ = 92%). As shown in Figure 3B, the treatment group resulted in a greater reduction in DHI than the control group (MD: 6.94; 95% CI: 1.58–12.30; *p* = 0.01). Sensitivity analysis revealed that heterogeneity decreased significantly (*p* = 0.60, *I*^2^ = 0%) after removing Wu’s study (26). It is possible that inconsistencies in the assessors’ criteria for judgment led to variability in their baseline and outcome levels thus resulting in clinical heterogeneity.

#### 3.5.3 THI

Three studies (22, 23, 26) compared the THI between groups, and the random-effects model was used because of the high heterogeneity among the studies (*p* < 0.0001, *I*^2^ = 95%). The result showed a greater reduction in THI of the treatment group than those of the control group (MD: 6.52; 95% CI: 0.77–12.27; *p* = 0.03) (Figure 3C). However, through sensitivity analysis, we were unable to find the source of heterogeneity, thus this result is unreliable.

#### 3.5.4 VAS

Three studies (22, 23, 26) compared VAS between groups. There was no significant heterogeneity among the studies in the change of VAS. (*p* = 0.70, *I*^2^ = 0%). The result showed a greater reduction in VAS of the treatment group than those of the control group (MD: 0.87; 95% CI: 0.54–1.20; *p* < 0.0001) (Figure 3D).

#### 3.5.5 Pure tone audiometry score

Three studies (22, 23, 26) compared pure tone audiometry score between groups. There was no significant heterogeneity among the studies in the change of pure tone audiometry score (*p* = 0.58, *I*^2^ = 0%). As shown in Figure 3E, the treatment group resulted in a greater reduction in pure tone audiometry score than the control group (MD: 6.57; 95% CI: 5.62–7.51; *p* < 0.0001).

#### 3.5.6 Adverse event

Only one RCT (23) reported adverse effects, and its two groups were safe and effective, none of the enrolled cases had adverse effects after treatment, and there was no obvious subcutaneous hemorrhage and local soft tissue injury.

### 3.6 Publication bias

**Figure 4:**
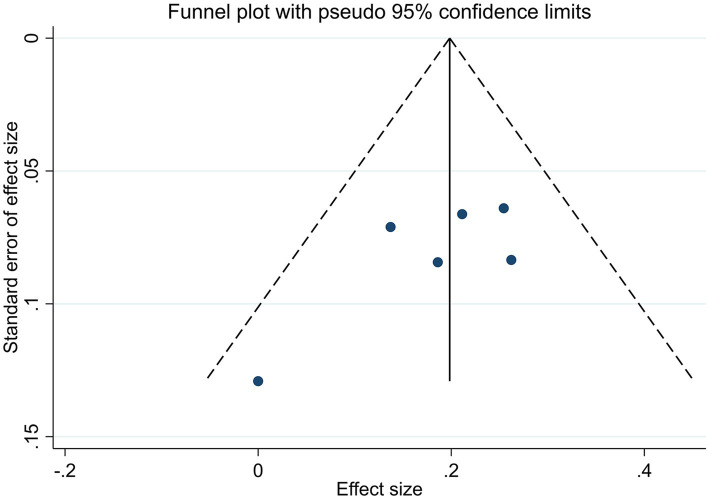
Funnel plot of efficacy rate.

In all studies that included efficacy rate, the funnel plots were symmetric (Figure 4). Egger’s test yielded a *p*-value of 0.144, indicating the absence of discernible publication bias (Supplementary material 5). Accordingly, the meta-analysis demonstrated that acupuncture was less susceptible to publication bias.

## 4 Discussion

A total of 6 RCTs with 494 (248/246) patients were included in our study to systematically evaluate the efficacy of acupuncture for MD. We found that the treatment group was superior to the control group with regard to efficacy rate. The treatment group resulted in a greater reduction in DHI, THI, VAS and pure tone audiometry score than the control group. However, due to the inability to identify the cause of heterogeneity, the results on THI should be considered unreliable.

A summary of the acupuncture protocols included in the study revealed that the most common acupoints were Baihui (GV20), Fengchi (GB20), Tinggong (SI19), Hegu (LI4), Quchi (LI11), Zusanli (ST36), Fenglong (ST40), Taichong (LR3), and Taixi (KI3). All trials used the uniform reinforcing-reducing needling method.

The pathology of MD is based on endolymphatic hydrops, which may be affected by various causes of excessive lymphatic fluid production or impaired absorption in the inner ear. In guinea pigs with endolymphatic hydrops, electroacupuncture was found to be able to increase the expression level of aquaporin 1 (AQP1) while also having a benign effect on serum ion concentration. This suggests that the improvement of endolymphatic hydrops by electroacupuncture may be related to the up-regulation of cochlear AQP1 expression and affected by the change in ion concentration (27). It was found that in the guinea pig model of AVP-induced endolymphatic hydrops, plasma AVP concentration was increased, cochlear V2R expression was downregulated, and cyclic adenosine monophosphate (cAMP) and aquaporin 2(AQP2) expression were upregulated. Electroacupuncture treatment reduced cochlear hydrops and reversed the expression of plasma arginine vasopressin (AVP), vasopressin type 2 receptor (V2R), cAMP, and AQP2 (28). Acupuncture at Baihui (GV20) was observed to reduce the degree of cochlear hydrops in the animal model of AVP-induced endolymphatic hydrops, accompanied by a down-regulation of cochlear AQP2 expression (29). Moxibustion at Tinggong (SI19) has been demonstrated to down-regulate plasma AVP, cochlear AQP2, and aquaporin 7(AQP7) expression in the animal model with endolymphatic hydrops, thereby achieving the effect of intervening in endolymphatic hydrops (30). The aforementioned findings supported the theory that acupuncture-mediated relief of endolymphatic hydrops and amelioration of clinical symptoms linked to MD may target the altered expression of inner ear water channel proteins in the model of hydrops. Our study has the following advantages. First, we used the updated and objective DHI, THI, VAS, and pure tone audiometry score to assess the severity of dizziness, tinnitus, hearing, and aural fullness in patients with MD. Secondly, three studies (22, 23, 26) had a low RoB, thus increasing the credibility of the findings. Finally, we restricted the type of control intervention used to reduce heterogeneity in the included studies and excluded Chinese herbs and Chinese Patent Medicine as co-treatment.

This systematic review and meta-analysis had several limitations. First, the credibility of our conclusions may have limitations due to the small sample size. Only six RCTs were included and the sample size was small, ranging from 30 to 58 patients, which may affect the precision and stability of the results. Second, three RCTs (21, 24, 25) did not describe the randomization method, and two RCTs (24, 25) had a high risk of bias regarding the measurement of outcome, all of which may affect the scientific reliability and validity of the RCTs. Third, all six studies (21–26) were conducted in China, which had regional and ethnic limitations. The culture, medical system, patient characteristics and other factors of different countries may affect the differences in the efficacy of acupuncture. Therefore, the location of all studies in China may affect the generalizability and external effect of the result. Similar studies should be conducted in other countries in the future. Fourth, the choice of acupuncture points, number, depth, retention time and type of needle suggest potential heterogeneity. Fifthly, only one RCT (22) reported a recurrence rate at 6-month follow-up (the treatment group 5.17% *VS* the control group18.97%). Due to other incomplete follow-up data, we were unable to compare the long-term effects of acupuncture for MD. Sixthly, only one RCT (23) reported adverse effects, which is an area for improvement in the future. Therefore, caution should be exercised when interpreting the results of this review.

In addition, some outcomes showed high heterogeneity, and sensitivity analyses were carried out to determine the source of heterogeneity. However, there is still high heterogeneity, possibly due to the following reasons. This is possibly related to differences in patient’s baseline levels, differences in interventions (acupoints selected, the manipulation of the performer, Western medicine), and differences in the measurement of outcome indicators by assessors.

Our meta-analysis indicates that acupuncture may have great potential for treating MD and warrants further investigation. The evidence base for acupuncture therapy in the treatment of MD is still limited. It is recommended that more large-scale, multi-center, rigorously designed, and conducted RCTs be carried out to provide the best evidence for clinical decision-making. Specific recommendations are as follows: Firstly, we should further evaluate the optimal acupuncture treatment protocol, acupuncture technique, efficacy criteria, and staging of MD for targeted treatment; Secondly, based on the current study (24), the minimum effective time for acupuncture treatment of MD maybe one week, and we need to further clarify the minimum effective time for acupuncture treatment of MD; Thirdly, we need to keep detailed records of follow-up data to assess the long-term efficacy of acupuncture for MD; Eventually, use high-quality clinical trial designs, such as RoB 2.0 and Consolidated Standards of Reporting Trials (CONSORT) guidelines (31), to optimize study design.

## 5 Conclusion

To sum up, acupuncture may improve the symptoms of vertigo, tinnitus, ear fullness and hearing loss in patients with MD, suggesting that acupuncture is a potential treatment for MD. However, due to the lack of literature included in this study and methodological weaknesses like randomization, blinding, and clinical heterogeneity, such as selected acupoints, acupuncture sessions, and therapist techniques more well-designed long-term follow-up RCTs are needed to evaluate the efficacy and safety of acupuncture.

## Data Availability

The original contributions presented in the study are included in the article/Supplementary material, further inquiries can be directed to the corresponding author.
