# Supplementary material for: Efficacy and safety of acupuncture in the treatment of Meniere’s disease: a systematic review and meta-analysis
Source: Front Med (Lausanne). 2024 Dec 11;11:1463821. doi: 10.3389/fmed.2024.1463821 (PMC11669254; doi:10.3389/fmed.2024.1463821)

Supplementary materials

[Supplement 1.Search strategy 1](#_Toc182689041)

[Supplement 2.STRICTA checklist 4](#_Toc182689042)

[Supplement 3.Acupoints of acupuncture groups 5](#_Toc182689043)

[Supplement 4.Details of needling 5](#_Toc182689044)

[Supplement 5.Egger's test 5](#_Toc182689045)

# Supplement 1.Search strategy

**From inception to June 1, 2024**

**PubMed**

#1Search: ((((((((((((meniere disease[MeSH Terms]) OR (Meniere Disease[Title/Abstract])) OR (Meniere[Title/Abstract])) OR (Menieres Disease[Title/Abstract])) OR (Meniere's Syndrome[Title/Abstract])) OR (Menieres Syndrome[Title/Abstract])) OR (Meniere Syndrome[Title/Abstract])) OR (Ménière Disease[Title/Abstract])) OR (Ménière Disease[Title/Abstract])) OR (Auditory Vertigo[Title/Abstract])) OR (Auditory Vertigos[Title/Abstract])) OR (Otogenic Vertigo[Title/Abstract])) OR (Otogenic Vertigos[Title/Abstract])

#2Search: (((((((((((((((acupuncture therapy[MeSH Terms]) OR (acupuncture therapy[Title/Abstract])) OR (acupuncture[Title/Abstract])) OR (acupoint[Title/Abstract])) OR (electroacupuncture[Title/Abstract])) OR (acupuncture treatment[Title/Abstract])) OR (electro-acupuncture[Title/Abstract])) OR (electric acupuncture[Title/Abstract])) OR (ear acupuncture[Title/Abstract])) OR (moxibustion[Title/Abstract])) OR (warm needling[Title/Abstract])) OR (fire needling[Title/Abstract])) OR (scalp acupuncture[Title/Abstract])) OR (auricular acupuncture[Title/Abstract])) OR (body acupuncture[Title/Abstract])) OR (plum blossom needle[Title/Abstract])

#3Search: ((((((((((randomized controlled trial[Publication Type]) OR (controlled clinical trial[Publication Type])) OR (randomized controlled trial[Title/Abstract])) OR (controlled clinical trial[Title/Abstract])) OR (RCT[Title/Abstract])) OR (randomized[Title/Abstract])) OR (clinical trials[Title/Abstract])) OR (placebo[Title/Abstract])) OR (randomly[Title/Abstract])) OR (trial[Title/Abstract])) OR (groups[Title/Abstract])

#1AND#2AND#3

N=5

**Web of Science**

#1 Search: (((((((((((TS=(meniere disease)) OR TS=(Meniere)) OR TS=(Menieres Disease)) OR TS=(Meniere's Syndrome)) OR TS=(Menieres Syndrome)) OR TS=(Meniere Syndrome)) OR TS=(Ménière Disease)) OR TS=(Ménière Disease)) OR TS=(Auditory Vertigo)) OR TS=(Auditory Vertigos)) OR TS=(Otogenic Vertigo)) OR TS=(Otogenic Vertigos)

#2 Search: ((((((((((((((TS=(acupuncture therapy)) OR TS=(acupuncture)) OR TS=(acupoint)) OR TS=(electroacupuncture)) OR TS=(acupuncture treatment)) OR TS=(electro-acupuncture)) OR TS=(electric acupuncture)) OR TS=(ear acupuncture)) OR TS=(moxibustion)) OR TS=(warm needling)) OR TS=(fire needling)) OR TS=(scalp acupuncture)) OR TS=(auricular acupuncture)) OR TS=(body acupuncture)) OR TS=(plum blossom needle)

#3 Search: ((((((((TS=(randomized controlled trial)) OR TS=(controlled clinical trial)) OR TS=(RCT)) OR TS=(randomized)) OR TS=(clinical trials)) OR TS=(placebo)) OR TS=(randomly)) OR TS=(trial)) OR TS=(groups)

#1AND#2AND#3

N=17

**EMBASE**

('meniere disease'/exp OR 'Meniere' OR 'Menieres Disease' OR 'Meniere's Syndrome' OR 'Menieres Syndrome' OR 'Meniere Syndrome' OR 'Ménière Disease' OR 'Ménière Disease' OR 'Auditory Vertigo' OR 'Auditory Vertigos'/exp OR 'Otogenic Vertigo' OR 'Otogenic Vertigos') AND ('acupuncture therapy' OR 'acupuncture' OR 'acupoint' OR 'electroacupuncture' OR 'acupuncture treatment' OR 'electro-acupuncture' OR 'electric acupuncture' OR 'ear acupuncture' OR 'moxibustion' OR 'warm needling' OR 'fire needling' OR 'scalp acupuncture' OR 'auricular acupuncture' OR 'body acupuncture' OR 'plum blossom needle') AND ('randomized controlled trial'/exp OR 'randomized controlled trial' OR 'controlled clinical trial'/exp OR 'controlled clinical trial' OR 'rct' OR 'randomized' OR 'clinical trials'/exp OR 'clinical trials' OR 'placebo'/exp OR 'placebo' OR 'randomly' OR 'trial'/exp OR 'trial' OR 'groups')

N=23

**Cochrane libraray**

#1 Search: (meniere disease):ti,ab,kw OR (Meniere):ti,ab,kw OR (Menieres Disease):ti,ab,kw OR (Meniere's Syndrome):ti,ab,kw OR (Menieres Syndrome):ti,ab,kw OR (Ménière Disease):ti,ab,kw OR (Auditory Vertigo):ti,ab,kw OR (Auditory Vertigos):ti,ab,kw OR (Otogenic Vertigo):ti,ab,kw OR (Otogenic Vertigos):ti,ab,kw

#2 Search: (acupuncture therapy):ti,ab,kw OR (acupuncture):ti,ab,kw OR (acupoint):ti,ab,kw OR (electroacupuncture):ti,ab,kw OR (acupuncture treatment):ti,ab,kw OR (electro-acupuncture):ti,ab,kw OR (electric acupuncture):ti,ab,kw OR (ear acupuncture):ti,ab,kw OR (moxibustion):ti,ab,kw OR (warm needling):ti,ab,kw OR (fire needling):ti,ab,kw OR (scalp acupuncture):ti,ab,kw OR (auricular acupuncture):ti,ab,kw OR (body acupuncture):ti,ab,kw OR (plum blossom needle):ti,ab,kw

#3 Search: (randomized controlled trial):ti,ab,kw OR (controlled clinical trial):ti,ab,kw OR (RCT):ti,ab,kw OR (Randomized):ti,ab,kw OR (clinical trials):ti,ab,kw OR (placebo):ti,ab,kw OR (randomly):ti,ab,kw OR (trial):ti,ab,kw OR (groups):ti,ab,kw

#1AND#2AND#3

N=13

**CNKI**

SU=（梅尼埃+美尼尔+内耳眩晕） AND SU=（针刺+针灸+针+灸+电针+火针+温针灸+埋线+耳针+耳穴+穴位+头针+腹针+体针） AND AB=（随机对照试验+随机对照研究+RCT+随机+对照）

N=37

**Wanfang**

主题:( 梅尼埃 or美尼尔 or 内耳眩晕 ) and 主题:(针刺 or 针灸 or 针 or 灸 or 电针 or 火针 or 温针灸 or 埋线 or 耳针 or 耳穴 or 穴位 or 头针 or 腹针 or 体针) and 摘要:(随机对照试验 or 随机对照研究 or RCT or 随机 or 对照 )

N=21

**VIP**

M=(梅尼埃 or 美尼尔 or 耳性眩晕) and M=(针刺 or 针灸 or 针 or 灸 or 电针 or 火针 or 温针灸 or 埋线 or 耳针 or 耳穴 or 穴位 or 头针 or 腹针 or 体针) and R= (随机对照试验 or 随机对照研究 or RCT or 随机 or 对照 )

N=41

**Sinomed**

("随机对照试验"[常用字段:智能] OR "随机对照研究"[常用字段:智能] OR "RCT"[常用字段:智能] OR "随机"[常用字段:智能] OR "对照"[常用字段:智能]) AND ("针刺"[常用字段:智能] OR "针灸"[常用字段:智能] OR "针"[常用字段:智能] OR "穴位"[常用字段:智能] OR "灸"[常用字段:智能] OR "电针"[常用字段:智能] OR "火针"[常用字段:智能] OR "温针灸"[常用字段:智能] OR "埋线"[常用字段:智能]) AND ("梅尼埃"[常用字段:智能] OR "美尼尔"[常用字段:智能] OR "耳性眩晕"[常用字段:智能])

N=83

# Supplement 2.STRICTA checklist

| **Item** | **Detail** | **Reported, % (n)** |
| --- | --- | --- |
| **1. Acupuncture rationale** | 1a) Style of acupuncture (e.g. Traditional Chinese Medicine, Japanese, Korean, Western medical, Five Element, ear acupuncture, etc) | 100% (6) |
|  | 1b) Reasoning for treatment provided, based on historical context, literature sources, and/or consensus methods, with references where appropriate | 100% (6) |
|  | 1c) Extent to which treatment was varied | 50% (3) |
| **2.**  **Details of needling** | 2a) Number of needle insertions per subject per session (mean and range where relevant) | 66.7% (4) |
|  | 2b) Names (or location if no standard name) of points used (uni/bilateral) | 100% (6) |
|  | 2c) Depth of insertion, based on a specified unit of measurement, or on a particular tissue level | 50% (3) |
|  | 2d) Response sought (e.g. *de qi* or muscle twitch response) | 100% (6) |
|  | 2e) Needle stimulation (e.g. manual, electrical) | 100% (6) |
|  | 2f) Needle retention time | 100% (6) |
|  | 2g) Needle type (diameter, length, and manufacturer or material) | 16.7% (1) |
| **3.**  **Treatment regimen** | 3a) Number of treatment sessions | 83.3% (5) |
|  | 3b) Frequency and duration of treatment sessions | 100% (6) |
| **4.**  **Other components of treatment** | 4a) Details of other interventions administered to the acupuncture group (e.g. moxibustion, cupping, herbs, exercises, lifestyle advice) | 100% (6) |
|  | 4b) Setting and context of treatment, including instructions to practitioners, and information and explanations to patients | 0% (0) |
| **5. Practitioner background** | 5) Description of participating acupuncturists (qualification or professional affiliation, years in acupuncture practice, other relevant experience) | 0% (0) |
| **6.**  **Control or comparator interventions** | 6a) Rationale for the control or comparator in the context of the research question, with sources that justify this choice | 100% (6) |
|  | 6b) Precise description of the control or comparator. If sham acupuncture or any other type of acupuncture-like control is used, provide details as for Items 1 to 3 above. | 100% (6) |

Note: This checklist, which should be read in conjunction with the explanations of the STRICTA items provided in the main text, is designed to replace CONSORT 2010’s item 5 when reporting an acupuncture trial.

# Supplement 3.Acupoints of acupuncture groups

| **Study, Year** | **Acupoint** |
| --- | --- |
| Ai 2013 | Taichong (LR3), Fengchi (GB20), Baihui (GV20), Sishencong(EX-HN1), Xingjian (LR2), Neiting (ST44), Xiaxi (GB43) |
| Ding 2021 | Baihui (GV20), Fengchi (GB20), Tinggong (SI19), Hegu (LI4), Quchi (LI11), Zusanli (ST36), Fenglong (ST40), Taichong (LR3), Taixi (KI3) and Tianshu (ST25) |
| Ha 2023 | Baihui (GV20), Tinggong (SI19), Ermen (SJ21), Tinghui (GB2), Hegu (LI4), Fenglong (ST40), Taixi (KI3), Sanyinjiao (SP6) |
| Mao 2014 | Baihui (GV20), Fengchi (GB20), Taichong (LR3), Neiguan (PC6), Tinggong (SI19), Wangu (GB12), Zusanli (ST36), Ganshu (BL18), Shenshu (BL23) |
| Wang 2011 | Baihui (GV20), Fengchi (GB20), Tinggong (SI19), Yifeng (SJ17), Taichong (LR3), Fenglong (ST40), Zusanli (ST36) |
| Wu 2018 | Baihui (GV20), Fengchi (GB20), Tinggong (SI19), Hegu (LI4), Quchi (LI11), Zusanli (ST36), Fenglong (ST40), Taichong (LR3), Taixi (KI3), Tianshu (ST25) |

# Supplement 4.Details of needling

| Number of needles | an average of 10.2 needles were used (range: 7–11) per session. The number of needles was either fixed (N = 3), or varied depending on the individual situation (N = 3) |
| --- | --- |
| Acupuncture points | body acupuncture points (N = 6). The most common acupoints were Baihui (GV20), Fengchi (GB20), Tinggong (SI19), Hegu (LI4), Quchi (LI11), Zusanli (ST36), Fenglong (ST40), Taichong (LR3), and Taixi (KI3) |
| Depth | the depth of needle insertion either followed TCM literature (N = 3), or not reported (N = 3) |
| Response | De Qi response was sought in all studies (N = 6) |
| Needle stimulation | needles were stimulated manually (N = 6) |
| Needle retention time | needles were left in place for 30 min (N = 4), or for 20 min (N = 2) |
| Needle type | sterile, disposable stainless-steel needles 0.18–0.3 × 25–40 mm (N = 3). Three study did not report the needle type used (N = 3) |

# Supplement 5.Egger's test


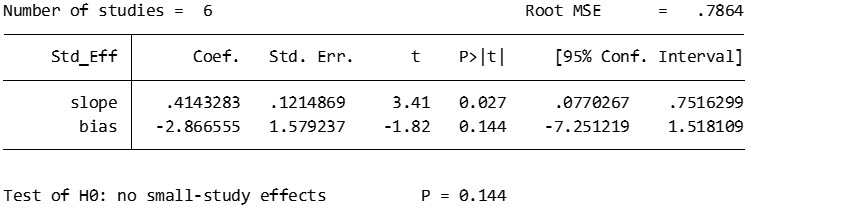

Supplement: Supplementary file 2 [file Data_Sheet_2.docx]
